# Beyond the Brain: Attention Deficit/Hyperactivity Disorder and the Gut-Brain Axis

**DOI:** 10.7759/cureus.76291

**Published:** 2024-12-23

**Authors:** Dhruv N Gandhi, Devina N Pande, Arya Harikrishna, Aditi Advilkar, Ishwar Basavan, Ramsha Ansari

**Affiliations:** 1 Internal Medicine, KJ Somaiya Medical College and Research Centre, Mumbai, IND; 2 Pediatrics, KJ Somaiya Medical College and Research Centre, Mumbai, IND; 3 Neurology, European University Cyprus - School of Medicine, Nicosia, CYP; 4 Internal Medicine, Jawaharlal Nehru Medical College, Belagavi, IND; 5 Internal Medicine, School of Medicine, Xiamen University, Xiamen, CHN

**Keywords:** adhd, attention-deficit/hyperactivity disorder, brain-gut interactions, faecal microbiota transplant, gut-brain axis, gut microbiome, gut microbiota, hyperactivity, impulsivity, probiotics

## Abstract

Attention-deficit/hyperactivity disorder (ADHD) is a complex neurodevelopmental condition, predominantly affecting children, characterized by inattention, hyperactivity, and impulsivity. A growing body of evidence has highlighted the potential influence of the gut microbiota on the onset and presentation of ADHD symptoms. The gut microbiota, a diverse microbial ecosystem residing within the gastrointestinal tract, exerts multiple effects on systemic physiology, including immune modulation, metabolic regulation, and neuronal signalling. The bidirectional gut-brain axis serves as a conduit for communication between gut microbes and the central nervous system, implicating its disruption in neurodevelopmental disorders such as ADHD. This comprehensive literature review aims to shed light on how alterations in the gut microbiota influence the development and manifestation of ADHD symptoms. Examining potential mechanisms involving gut microbial metabolites and their impact on neurotransmitter modulation, neuro-endocrine signalling and neuroinflammation, we dissect the intricate interplay shaping ADHD pathology. Insights into these complex interactions hold promise for personalized therapeutic interventions aimed at modulating the gut microbiota to ameliorate ADHD symptoms. Discussions encompass dietary interventions, faecal microbiota-targeted therapies, and emerging probiotic approaches, underscoring their potential as adjunctive or alternative strategies in managing ADHD. Further research elucidating the precise mechanisms driving these interactions may pave the way for targeted and personalized interventions for individuals grappling with ADHD.

## Introduction and background

Attention-deficit/hyperactivity disorder (ADHD) is a multifaceted neurodevelopmental condition that has drawn attention from paediatricians, neurologists and psychiatrists ever since its discovery. As the name suggests, it is characterized by inattention, hyperactivity, and impulsivity [1]. However, the underlying aetiology behind this seemingly innocuous symptomatology remains complex, with research exploring novel factors influencing its onset and progression. Among these factors, the role of the gut microbiota has gained substantial attention in the last few years.

The gut microbiota refers to the diverse community of microorganisms naturally residing within the gastrointestinal (GI) tract, playing a pivotal role in various physiological functions. The gut microbiota mainly consists of six phyla of Bacteroides, Firmicutes, Actinomycetes, Verrucomicrobia, Fusobacteria and Proteobacteria with Firmicutes and Bacteroides being the predominant phyla [2]. They aid in the digestion and absorption of nutrients such as carbohydrates and proteins, as well as vitamins, bile acids and other bioactive molecules [3]. Gut microbiota also plays a profound role in brain development and neuroendocrine functioning [4-7].

One particular interest is the gut-brain axis (GBA), a bidirectional communication pathway linking the gut and the central nervous system (CNS). These intricate connections involve neural, endocrine and immune pathways, allowing constant interaction between the gut microbiota and the brain at a molecular and cellular level [8]. In neurodevelopmental conditions like ADHD, disruptions in this axis are believed to contribute significantly to the condition's pathophysiology [9].

Understanding the influence of alterations in gut microbiota in the development and symptomatology of ADHD represents a crucial avenue of research. The interplay between gut microbes and the CNS raises compelling questions regarding their role in neurotransmitter function, neuronal inflammation and nervous signalling, all of which are implicated in ADHD [9].

Thus, the main research question posed is: "How do gut microbiota influence the development and symptomatology of ADHD?" We delve into the mechanisms by which the composition and diversity of gut microbes potentially modulate brain development, cognitive processes, and behavioural patterns associated with ADHD. Exploring this relationship holds promising implications for developing personalised therapeutic interventions and novel dietary strategies aimed at modulating the gut microbiota in order to mitigate ADHD symptoms. 

## Review

ADHD and the gut: an emerging connection 

ADHD is a disorder affecting individuals worldwide, although the prevalence and incidence rates have been highly variable [10,11]. Due to the variability, various meta-analyses have been conducted to understand the cause of this variation [12,13]. One such important study involving children and adolescents from 35 countries estimated the prevalence of ADHD to be 5.3% [14]. Follow-up meta-regression analyses showed that the variability in previous studies was due to methodological differences (e.g., diagnostic criteria) between the studies [11,13]. ADHD prevalence in adults (19-45 years) is estimated to be at 2.5% worldwide [14]. However, the prevalence has been increasing in the past 20 years [15].

ADHD is mainly characterized by inattention, hyperactivity, and impulsivity [12,16]. Children with ADHD have an increased risk of accidental injuries, worse quality of life, impaired school performance, poor relationships with parents and peers, and emotional dysregulation [14,17]. Adolescents with ADHD are also at higher risk of substance abuse, earlier participation in sexual activities, and more frequent teenage pregnancies [17]. Adults with persistent ADHD have shown lower job performance, increased emotional problems, higher risk of road traffic accidents, criminality, unemployment, and substance abuse [17]. Due to the high burden of the disease, improper treatment can result in poor quality of life and undesired outcomes such as career and/or academic failures, unwanted accidents, and poor interpersonal relationships [18]. Hence, it is important to understand the aetiology of ADHD to identify the severity of the disease and start timely management/treatment [19]. 

Despite ADHD being fairly common worldwide, the aetiology remains unclear [20]. Genetic influences, environmental exposures, pre-, peri-, and postnatal factors, and social factors have all been implicated in the development of ADHD [12]. ADHD is highly heritable with most estimates ranging from 70 to 80% [21]. There are 12 genome-wide significant loci that have been identified through genome studies [12]. Prematurity and low birthweight have been persistently associated with ADHD even when there are no genetic confounders [22-24]. Understanding the aetiology of ADHD can aid in providing early interventions which could potentially increase their efficacy and result in a better quality of life [25]. 

The literature on psychiatric comorbidities in ADHD has been well documented, but its association with somatic disease is limited [26]. Currently, obesity, asthma, sleep disorders, otitis media, urinary symptoms, migraine, celiac disease, allergic rhinitis, and motor disturbances have all been implicated in ADHD [26-28]. Very few studies explore GI comorbidities in adults with ADHD. One such study revealed that the ADHD group had more cases of dyspepsia, chronic constipation, and irritable bowel syndrome (IBS); compared to the placebo group [26]. Although more research data are available in this regard for children, the reported results are inconsistent [29]. Few studies reported increased ADHD prevalence in children with constipation, chronic diarrhoea, IBS, and encopresis [30,31], whereas some reported higher rates of abdominal pain, abdominal distention, obesity, and food allergy in children with ADHD [30-34]. Other studies found no association at all between ADHD and GI symptoms [12,35]. 

Recently, the gut microbiome has been identified as a factor associated with ADHD [36]. The GBA is a widely studied system currently due to its influence on the brain activity (and vice versa) via the gut microbiota environment. This bidirectional communication includes the CNS, autonomic nervous system (ANS), enteric nervous system (ENS), and hypothalamic-pituitary-adrenal axis (HPA) [18]. The gut flora is said to be involved in immune regulation [37]. Hence, any imbalances in the microbiota (also known as dysbiosis) can result in immune dysregulation and in turn systemic inflammation, which is believed to be associated with ADHD [38,39]. 

The gut microbiota-brain axis: mechanisms of interaction

**Figure 1:**
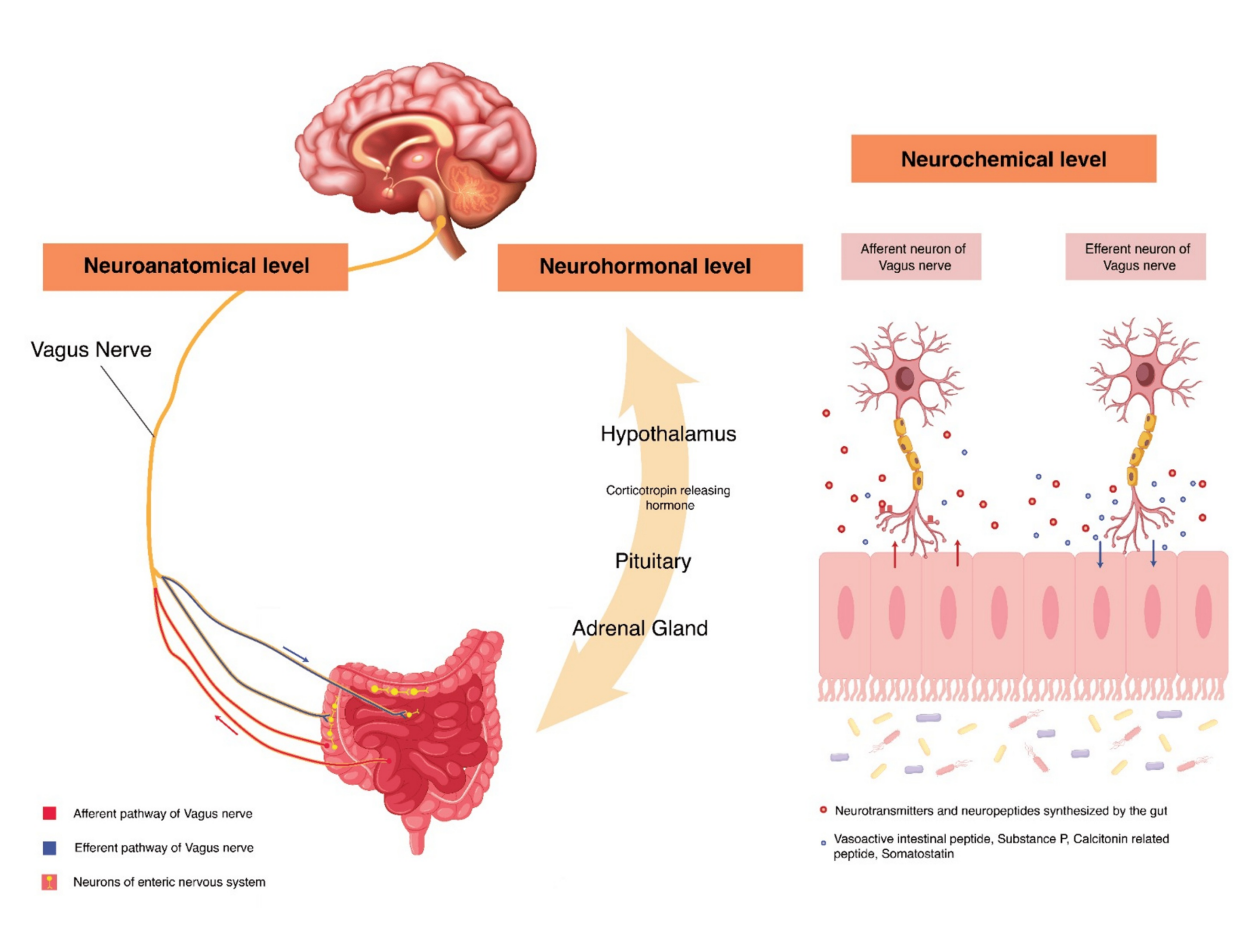
Pathways of Gut-Brain Communication at Various Levels Levels of communication between the gut and the brain: the neuroanatomical, neurohormonal and neurochemical levels (Attribution: Aditi Advilkar).

The GBA was first hypothesized in the 19th and early 20th century and has been the subject of increased research over the last few decades [40,41]. The GBA involves the GI tract, CNS, ENS, ANS, and HPA. The precise mechanism in which the gut microbiome influences the brain function is not fully understood. However, there are a few potential pathways through which this complex communication occurs [42], as shown in Figure 1. The GBA links emotional/cognitive centres of the brain with peripheral intestinal functions (entero-endocrine, immune activation, enteric reflex, and intestinal permeability) [8]. The GBA also monitors and regulates gut microbiota via neuroanatomical, neurohormonal, and immunological pathways and communicates via neuropeptides, neurotransmitters, and microbial-derived products [43].

The neuroanatomical pathway comprises two communication paths, (i) via the vagus nerve (VN) and ANS in the spinal cord (SC) and (ii) ENS within the gut and ANS plus VN within the SC [44]. Signals from the gut microbiota are carried by the enteric neurons and the vagus nerve to higher centres in the brainstem, basal ganglia, thalamus, limbic system and insular cortex [8,43]. This is of significance because the microbiota can induce a signal through the VN and vice versa. Recognizing the early interactions between ADHD and gut microbiota could possibly be of high value as it can open new interventional strategies along with early management. The bidirectional feedback loop is closed by regulatory signals from the brain via the ENS and the VN to influence gut motility, hormonal secretion, immune function and permeability [8,43]. 

Another level of interaction is via the neurohormonal pathway. Gut microbiota have been shown to modulate the stress response through regulation of the HPA axis. Initially, corticotropin-releasing hormone (CRH) is released by the hypothalamus under stress, which further triggers the release of adrenocorticotropic hormone (ACTH) from the pituitary gland. ACTH induces the release of glucocorticoids, mineralocorticoids, and catecholamines from the adrenal cortex, which modulates the gut microbiota composition, intestinal permeability and immune regulation [45]. It is also worth noting that ACTH not only acts on the adrenal cortex but also influences brain functions/behaviours. This links the neurohormonal pathway with the neurochemical pathway and that ACTH or other neuropeptides can serve multiple purposes [46].

At the neurochemical level, the gut microbiota synthesizes several neurotransmitters, neuropeptides, and microbial-derived products such as acetylcholine, histamine, serotonin, melatonin, dopamine, CRH, gamma-aminobutyric acid (GABA), and short chain fatty acids (SCFAs). These neurochemical components aid in modulating internal connections within the nervous system and external connections with the endocrine/immune system [43]. Another important factor is an intact blood-brain barrier (BBB), as it has been theorized that the gut microbiota could be regulating the integrity of the BBB. For instance, SCFAs produced by bacterial fermentation can regulate permeability of the BBB, mucosal serotonin secretion and microglial maturation [47]. They can also influence the expression of brain derived neurotrophic factor (BDNF), serotonin receptors and N-methyl-D-aspartic acid receptors in the hippocampus and cortex [43]. In return, vasoactive intestinal peptide, substance P, calcitonin gene related peptide (CGRP) and somatostatin influence gut microbiota functions [43].

GABA has been found to be synthesized in the human gut by Bacteroides fragilis, Eubacterium, Bifidobacterium and Parabacteroides [48]. However, peripherally synthesized GABA cannot cross the BBB suggesting that GABA modulates neurological functions through the VN or ENS [49]. Similarly, glutamate can also be synthesized by the gut microbiota and cannot cross the BBB. Glutamate most likely modulates neurological functions through the ENS and VN [49]. Metabolites of carbohydrate fermentation in the colon such as SCFAs can cross the BBB and get incorporated into the metabolic cycles of glutamate-glutamine-GABA in the hypothalamus [50]. Low concentrations of GABA, glutamine and glutamate have been found in adults with ADHD [51,52].

The GI tract is said to be an important site for serotonin biosynthesis. It has been found that the Clostridial species in the gut can increase the synthesis of serotonin by upregulating tryptophan hydroxylase 1 in enterochromaffin cells through intermediate signalling molecules [53]. Another study found serotonin production in Staphylococci via staphylococcal aromatic acid decarboxylase (SadA) enzyme [54]. Serotonin cannot cross the BBB by itself; however, tryptophan which is synthesized by the bacterial kynurenine pathway can cross the BBB [55]. Low levels of serotonin in the brain have been associated with ADHD [56]. Dopamine can be produced by Staphylococci within the gut from its precursor L-dopa by the enzyme SadA [54]. It is worth noting that dopamine dysregulation is associated with altered reward pathways in young children with ADHD [57,58].

Animal studies: gut microbiota and ADHD 

Intestinal bacterial colonization modulates the host metabolome, which influences CNS function [59,60]. The GBA has garnered recognition, underscoring the central role of the gut microbiota in influencing behaviour and cognitive function. Research utilizing animal models has consistently indicated the microbiota's crucial involvement in foundational neural processes, including but not limited to neurogenesis, myelination, and microglia activation [61].

The intestinal microbiota exerts an impact on brain chemistry and behaviour, demonstrating independence from the autonomic nervous system, gastrointestinal-specific neurotransmitters, or inflammation. This suggests that disturbances in intestinal microbiota could potentially contribute to psychiatric disorders [62]. Extensive research conducted by Fung et al. [63] and Lukić et al. [64] provides substantive evidence elucidating the profound effects of gut microbiota on both the immune and nervous systems [63,64]. Changes in the microbial composition have been linked to variations in behaviour and cognitive functions and animal studies have played a crucial role in elucidating this complex relationship [65].

In a study conducted by Tengeler et al. [66], the researchers transplanted gut microbiota from individuals with and without ADHD into germ-free (GF) mice, resulting in distinct microbiome profiles. The mice colonized with ADHD microbiota displayed altered brain structure, reduced integrity in white and grey matter regions, and disrupted functional connectivity in brain areas associated with neurodevelopmental disorders. Moreover, these mice exhibited heightened anxiety levels in behavioural tests. These findings collectively underscore the substantial impact of gut microbiota on both brain structure and behaviour in the context of ADHD, as revealed in their research [66].

Studies by Desbonnet et al. [67] and Kelly et al. [68] have investigated the impact of probiotics on animal behaviour. Desbonnet et al. observed that the probiotic Bifidobacterium infantis displayed antidepressant effects in a rat model. This outcome underscores the potential therapeutic significance of manipulating the gut microbiota in psychiatric conditions, such as ADHD [67]. Kelly et al. [68] found that transplantation of faecal microbiota from depressed individuals to microbiota-depleted rats led to anxious behaviours and changes in tryptophan metabolism. The manipulation of the gut microbiota through dietary interventions induces diet-dependent comprehensive alterations in white matter structural integrity [69].

In their study, Lach et al. [70] explored the impact of gut peptides on anxiety and depression, highlighting the concept that gut microbiota can exert a direct influence on emotional well-being. Sgritta et al. [71] showcased the correlation between alterations in social behaviour mediated by microbes and their association with autism spectrum disorder in mouse models. This implies that the influence of the gut microbiota on neurodevelopmental conditions such as ADHD extends beyond individual behavioural effects to encompass broader societal and interpersonal repercussions [71].

The gut-brain interaction suggests that the gut microbiota may have the ability to impact neurotransmitter systems within the brain, potentially playing a role in the manifestation of ADHD symptoms. This concept finds support in the investigations conducted by Zheng et al. [72], who demonstrated that modifications of the gut microbiome resulted in a depressive-like behaviour in an animal model. Additionally, the discussions put forth by Vuong and Hsiao [73] shed light on the evolving roles of the gut microbiome in autism spectrum disorder, underlining the significant implications of the GBA in the context of neurodevelopmental disorders.

An assessment of activity and anxiety in adult GF mice and those with a normal gut microbiota (SPF) using an open-field activity box was done by Heijtz et al. [74]. GF mice exhibited increased total distance travelled and greater exploration of the centre of the open field compared to SPF mice (P < 0.05). Although both groups initially displayed similar locomotor activity, GF mice demonstrated significant differences over time, traveling a longer distance and spending more time in both slow and fast locomotion during the 20- to 60-min interval (P < 0.05). Additionally, there was a trend for higher rearing activity in GF mice (P = 0.088) [74].

Human studies: gut microbiota and ADHD

Human studies have revealed fascinating insights into the interplay between gut microbiota and ADHD symptomatology and disease progression. A collection of diverse studies across varied populations, ranging from Chinese children to Dutch adolescents to Spanish adults, has shed light on the relationship between specific gut microbial compositions and ADHD symptoms. These studies have revealed differences in gut microbial profiles, particularly in genera like Bifidobacterium, Ruminococcaceae, Faecalibacterium, and Bacteroides, among others, implicating their potential roles in ADHD. These findings, often influenced by dietary patterns and demographic disparities, highlight the impact of the GBA on ADHD pathogenesis.

Aarts et al. [75] and Szopinska-Topov et al. [76] conducted studies on Dutch adolescents and young adults comparing the gut microbiome composition of ADHD patients with that of healthy controls. Aarts et al. [75] chose 96 Dutch adolescents and young adults, of which 19 had ADHD and 77 were healthy controls while Szopinska-Topov et al. [76] chose 107 participants into one of three groups: 42 participants who met the criteria for ADHD, 15 participants who did not meet the criteria for ADHD but scored high enough to not be considered as healthy controls and 50 healthy controls.

Aarts et al. [75] found a significant increase in Actinobacteria (ADHD: 22% to controls:14%) which seemed to be compensated by a decrease in Firmicutes (ADHD: 70% to controls: 80%) while the rest of the microbiome did not differ significantly between the two groups. Within phylum Actinobacteria, the genus Bifidobacterium was found to be significantly increased within the ADHD group (ADHD: 20.50% to controls: 12.67%). Due to the decrease in the relative prevalence of Bifidobacterium in the gut with ageing, an age-matched subsample was tested and interestingly, results following age-matching also showed a similar trend [75].

In contrast to Aarts et al. [75], Szopinska-Topov et al. [76] found no significant differences in the relative prevalence of Actinobacteria and Firmicutes between the two groups. However, they did find a significant increase in the genus Ruminococcaceae_UGC_004, which was associated with inattention symptoms. A drawback of Aarts et al. [75] was that they did not consider ADHD medication as a potential confounder. This was addressed by Szopinska-Topov et al. [76] who found that ADHD medication did not affect the association between genus Ruminococcaceae_UGC_004 and inattention symptoms. They also found no association between the isolated genus and impulsivity/hyperactivity symptoms [76].

The relative increase in Bifidobacterium was correlated with increased levels of cyclohexadienyl dehydratase (CDT), which is involved in the formation of dopamine precursors. The relative abundance of CDT was found to be negatively correlated with reward anticipation in the ventral striatum bilaterally. This decrease in reward anticipation is a characteristic finding in ADHD [75]. On genomic analysis, it was found that Ruminococcaceae_UGC_004 had similar sequences to certain bacteria which had the ability to consume GABA [76]. Low levels of GABA, an inhibitory neurotransmitter, have been found to be associated with ADHD [51]. 

The findings from the study conducted by Jiang et al. [77] on a Chinese population of 51 treatment-naive ADHD children and 32 age-matched healthy controls, contrasted from those obtained by Aarts et al. [75] and Szopinska-Topov et al. [76]. They found that the genus Faecalibacterium of the family Ruminococcaceae had a lower prevalence in the ADHD group. While Ruminococcaceae_UGC_004 is associated with inattention symptoms [76], low levels of Faecalibacterium have been associated with higher Conners Parent Rating Scales values as well as higher hyperactivity index scores [77]. These differences in the gut microbiota composition may be attributed to racial differences as well as differences in the Chinese diet and the high-fat Western diet respectively [75-77]. This study also took into account potential confounders such as the effect of ADHD medication, the use of antibiotics and probiotics, the presence of atopy and the presence of depressive, anxiety or GI symptoms [77].

Wan et al. [78] conducted a case-control study similar to that by Jiang et al. [77] in terms of the study population and the control of potential confounders; however, it entailed a smaller population of cases and controls. They corroborated the finding of decreased Faecalibacterium in the ADHD group [77,78]. Faecalibacterium has been found to exert anti-inflammatory effects and the reduction of Faecalibacterium has been hypothesized to increase inflammatory cytokine levels leading to neuronal dysregulation in ADHD [77,78]. In contrast to Jiang et al. [77], Wan et al. [78] found significantly increased Odoribacter and Enterococcus in the ADHD group. The increase in Enterococcus is hypothesised to reduce intracranial dopamine and contribute to ADHD pathogenesis [78].

Wang et al. [79] and Jung et al. [80] found that differences in dietary patterns may contribute to differences in gut isolates, further suggesting that diet can influence gut microbiota composition in patients of ADHD [77-80]. Wang et al. [79] examined the food habits and faecal microbiota compositions of 30 Taiwanese ADHD children and 30 healthy controls while Jung et al. [80] assessed the same in 40 Korean elementary school students. In addition, Jung et al. [80] also measured the faecal SCFA concentration.

Wang et al. [79] found that Bacteroides uniformis, Bacteroides ovatus and Sutterella stercoricannis were significantly more abundant in the ADHD group while the relative prevalence of Bacteroides coprocola was significantly lower. There was a correlation found between the amount of S. stercoricannis and the consumption of dairy, ferritin, nuts, seeds and legumes. While B. ovatus and B. coprocola showed no relationships with any of the items in the food frequency questionnaire, B. uniformis was linked to the intake of fat and carbohydrates. There is a favourable correlation between S. stercoricannis and B. ovatus with symptoms of ADHD [79]. 

Jung et al. [80], on the other hand, found that children with ADHD in the processed food group were more likely to have isolates of Escherichia coli, Enterobacter and Clostridium and were less likely to have isolates of Bifidobacterium and Ruminococcus. These findings contrast from the findings of Aarts et al. [75] and Szopinska-Topov et al. [76] while they correlate with Jiang et al. [77], suggesting that Asian diets were likely to influence gut isolates in patients of ADHD. Jung et al. [80] also found a significant negative correlation between the SCFA concentration in feces and the ADHD score, suggesting that altered colonic flora may contribute to ADHD pathogenesis.

Prehn-Kristensen et al. [81] examined the variations in the microbiome of a group of 14 German teenagers with ADHD and 17 healthy controls. There were no dietary variations between the two groups. Neisseria was shown to be an indicator for the ADHD group, while Prevotella and Parabacteroides were found to be indicators for the control group. This study found that while there was no difference in alpha diversity between the fathers of ADHD patients and controls, there was in the mothers of ADHD patients. Thus, it raises the possibility that changes in the composition of the microbiome may be transferred from mothers to their offspring [81].

The study by Richarte et al. [82] is unique in that it compared the gut microbiota composition of 100 Spanish adult ADHD cases with 100 healthy controls, while all previous studies were based on paediatric populations. The potential confounding effect of ADHD medication, antibiotics and probiotics was excluded in this study. The ADHD group had reduced abundances of Gracilibacter and Anaerotaenial and greater levels of Megamonas and Dialister in comparison to the control group. Toddler activity level and Dialister abundance have been shown to positively correlate. When compared to healthy controls, lower levels of Dialister were seen in patients with autism spectrum disorder (ASD), in treatment-naïve children with ADHD, and in ADHD-treated patients compared to untreated patients [82].

Cheng et al. [83] used gene set enrichment analysis (GSEA) to investigate possible connections between the gut microbiota and five diseases: major depressive disorder (MDD), ADHD, ASD, bipolar disorder (BD), and schizophrenia (SCZ). The Psychiatric GWAS Consortium's publicly accessible genome-wide association studies (GWAS) provided the data for GSEA. For the ADHD dataset, they took 19,099 ADHD patients and 34,149 healthy controls. They found a significant association between ADHD and Clostridiales order and Desulfovibrio genus. However, Desulfovibrio was also associated with MDD, BD, and SCZ and most strongly with ASD [83].

To summarise, the gut microbiota in ADHD patients is associated with an increase in the prevalence of B. uniformis, B. ovatus, Odoribacter, Enterococcus, E. coli, Enterobacter, Clostridium, S. stercoricannis, Neisseria, Megamonas, Dialister and Desulfovibrio. On the other hand, decreased prevalence of Faecalibacterium, B. coprocola, Prevotella, Parabacteroides, Gracilibacter and Anaerotaenia has been associated with the gut microbiome of ADHD patients. The prevalence of Bifidobacterium and Ruminococcus is conflicting, with an increased prevalence reported in one study while a decreased prevalence in ADHD patients reported in another study [75-82].

Potential therapeutic interventions 

The approach towards the management of neurodegenerative and neurodevelopmental disorders is often multidimensional due to the absence of standard protocols. Clinicians have conventionally used pharmacologic agents such as stimulants and antipsychotics with psychosocial interventions such as behavioural therapy in the management of ADHD. These agents, while offering symptomatic relief, have been shown to cause multiple adverse effects, leading to the exploration of other potential therapies [84]. Therapies involving the modulation of gut microbiota have gained attention in the recent past due to growing evidence of a link between the GBA and ADHD. Some of the key strategies include dietary and lifestyle modifications, probiotics, antibiotics and faecal microbiota transplantation.

Diet plays a crucial role in modulating gut microbiota. Evidence suggests that dietary habits exert a substantial influence on the likelihood of the development or exacerbation of symptoms of ADHD, with unhealthy eating exhibiting a positive correlation with the disease. In a study conducted in Korea involving 986 school-age children with ADHD and learning disabilities, the researchers observed a positive association between a high consumption of sweetened desserts, fried foods, and salt, and the prevalence of learning, attention, and behavioural problems. Conversely, they found that maintaining a balanced diet, which included regular meals, good intake of dairy products and vegetables, was negatively associated with these issues [85-87]. The findings of another study also suggested that addressing and managing dietary and nutritional factors may be a consideration for alleviating ADHD and its subtypes in school-age children. They found that iron, vegetable protein, and calcium demonstrated inhibitory effects on ADHD [88]. Furthermore, disturbances in the levels of essential nutrients like vitamin D, iron, zinc, and polyunsaturated fatty acids (PUFAs) have also been linked to the exacerbation and progression of ADHD [86,87]. Yang et al. found that lower than normal serum 25-hydroxy cholecalciferol levels may act as a potential contributing factor to the upregulation of attention deficit and hyperactivity disorder (ADHD) expression in certain school-age children [89]. Elshorbagy et al. conducted a study focusing on children with cognitive disabilities and vitamin D deficiency, exploring the impact of vitamin D supplementation on these individuals. The findings revealed positive results, indicating an improvement in cognitive function following the administration of vitamin D supplements [90,91].

In the year 1973, Dr. Ben Feingold recommended a diet, now known as the Feingold diet, free of salicylates, artificial flavours, and artificial food colours that he believed caused hyperactivity. Adoption of this diet resulted in an improvement in the behaviours of many children [92]. Pessler et al. investigated the effect of a few-foods diet on ADHD since some studies provided evidence that certain foods can trigger ADHD. A double blinded placebo trial was conducted and it was found that identifying and removing the implicated foods, and underlying causal triggers, could contribute to preventing instances of food-induced ADHD [93]. A pilot study investigated the effect of administration of micronutrients on faecal microbiome content and provided findings that revealed an increase in bacteria from the Bifidobacterium genus. Although further research is required to establish the exact role of this genus in ADHD, micronutrient administration may be used in the future as a therapeutic intervention [94].

Psychobiotics include a group of probiotics that modulate the activation of the VN by influencing neurotransmitters such as GABA, serotonin, and glutamate, along with proteins such as BDNF. These are pivotal in maintaining the equilibrium between neural excitation and inhibition and impacting mood, anxiety, cognitive functions, learning, memory processes, and even appetite enhancement [85]. Studies evaluating these probiotics through various randomized controlled trials (RCTs) indicate that several lactobacillus strains including L. Paracasei 37, L. Planetarium 128, L. reuteri DSM 17938, and Bifidobacterium longum have been effective at improving symptoms of ADHD. These include disruptive antisocial behaviours, anxiety, communication, adaptive functioning, sensitivity biographies, and hyperactivity [85]. An RCT conducted to study the effect of Lactobacillus rhamnosus GG ATCC53103 (LGG) also came to the conclusion that children and adolescents with ADHD who received supplements of this strain reported better functioning physically, emotionally, socially and scholastically [95]. In addition to the use of probiotics ADHD post-diagnosis, studies have also found that supplementation of Lactobacillus rhamnosus GG early in life may be preventive in nature. It has been shown to stabilize the gut permeability barrier by its effects on tight junctions, mucin production and antigen-specific immunoglobulin A production, thereby modulating the development of ADHD [96]. 

Skott et al. [97] studied the effects of Synbiotic 2000 in children and adults with ADHD who did not have an autism diagnosis in a placebo-controlled double-blind RCT. Synbiotic 2000 features a combination of three lactic acid bacteria with anti-inflammatory properties, and four fibre sources with anti-inflammatory benefits. Although no distinct Synbiotic 2000-specific effect was identified, an examination of individuals exhibiting elevated plasma sVCAM-1 levels suggested an enhancement of emotion regulation in adults with ADHD [97].

Faecal microbiota transplantation (FMT) is an intervention to correct the imbalance of the gut microbiome and potentially reintroduce absent microorganisms via the transfer of a healthy donor’s faecal specimen to the GI tract of a recipient patient [98,99]. This may in turn boost the production of hormones and neurotransmitters that play a vital role in regulating human behaviour and cognition. A 22-year-old woman treated with FMT for recurrent Clostridioides difficile infections was coincidentally relieved from her ADHD symptoms. This provided preliminary evidence for the use of FMT in the management of ADHD [98]. Various bacteria engrafted through FMT have shown to have a positive impact on the alleviation of ADHD symptoms. These include F. prausnitzii which may increase anti-inflammatory cytokines and decrease inflammatory cytokines that promote the development of ADHD. L. ruminis may also be neuroprotective in nature by producing anti-inflammatory SCFAs [99].

Although more research is needed to evaluate the efficacy of these interventions, the use of gut modulating therapies seems promising in the management of ADHD.

Methodological challenges and future directions 

Alternative novel therapies modulating gut microbiota are accompanied by their fair share of challenges. The pooled analysis of 14 observational studies (four cohorts, five case-control and five cross-sectional studies) found that healthy dietary patterns were protective against ADHD (OR: 0.65; 95% CI: 044 - 0.97), while unhealthy dietary patterns were found as risk to ADHD (OR: 1.41; 95% CI: 1.15-1.74) [70]. Concurrently, Ríos-Hernández et al. [100] found that children with ADHD show less adherence to healthy eating patterns than children without this disorder. Thus, the observational studies suggest a possible role for dietary patterns in ADHD; however, causal relationships between diet and ADHD cannot be established [85].

The effects of dietary supplements have been reported in some studies, but severe methodological limitations have been identified, including short intervention periods, lack of randomization, placebos, and prospective monitoring of long-term effects. From a clinical standpoint, these diets must be used with caution as restriction and elimination diets can lead to nutritional deficiencies in children with ADHD, so their nutritional status must be monitored rigorously [101]. Even though the administration of micronutrients to faecal microbiome content revealed an increase in bacteria from the Bifidobacterium genus [94], the connection between Bifidobacterium and its effect on ADHD symptoms still needs to be analyzed. At the same time, further studies are essential to learn more about pro-inflammatory cytokines regulated by gut microbiota [18].

In addition to the existing therapies, psychobiotics remain promising, although it is possible that their efficacy will diminish over time due to environmental factors having a significant influence on child development [99]. More research is still needed for deeper understanding of psychobiotics, their formulation, strain, dosage, timings, the effect of different strain combinations and country-specific variations, thereby offering enduring relief from core symptoms along with quality-of-life improvement in patients with ADHD [84]. Probiotic supplementation early in life and its role along with mechanisms in reducing the risk of ADHD in later life should also be explored [96]. Additionally, the novel therapeutic functions of the psychobiotics and commensal bacteria should be investigated in synthetic biology fields.

Although the RCT conducted to study the effect of Lactobacillus rhamnosus GG concluded that children with ADHD who received the supplementation of this strain reported better overall functioning, a longer observation period (6-12 months), inclusion of more children's self-report assessments and recruitment of non-drug naive patients would give a clearer picture [95].

The actual efficacy of FMT has also been proven by various studies using diverse animal models. A colon-release capsule coated with acid-resistant hydroxypropyl cellulose is the best formulation for patients. However, since most ADHD patients are children, safety becomes the most crucial aspect. In previous studies, the oral ingestion of human faecal suspensions was considered an unpleasant experience for patients. Additionally, it might cause side effects, particularly due to acid suppressants which usually accompany FMT capsules [99].

Numerous studies demonstrate that early alterations in the gut microbiome are closely related to neurodevelopmental disorders, such as ADHD. Intestinal microbiota colonization and establishment in early stages of life are profoundly affected by maternal conditions and diseases, delivery mode, and antibiotic exposure. It is therefore necessary to conduct further studies in order to determine precise therapeutic targets in immune, metabolic, endocrine, and neural pathways by validating mechanisms through culturomics experiments involving primarily modulated microbial populations, as well as metabolomic analysis of the skin, vagina, gut microbiota, and infant gut environments [99].

In order to better comprehend the crucial role of the gut microbiome in ADHD pathogenesis, extensive double-blind, RCTs are recommended. At the same time, treatments tailored to individual characteristics and the host microbiome should be investigated. 

To summarize, the gut microbiota's role in ADHD is still poorly defined because of a) varied study designs, b) small number of patients, c) different age groups analyzed, d) including only treatment naive patients versus those receiving medication, e) differences between males and females, and f) heterogeneous technologies used to analyze microbiome sequences.

## Conclusions

ADHD is a globally prevalent disorder with significant morbidity. Evidence from studies suggests the potential role of bidirectional communication between the gut microbiome and the host's brain in the development of ADHD. Several studies highlight the complexity of this relationship, influenced by factors like diet, medication, and potential maternal transmission. These have provided a basis for exploration of therapeutic strategies aimed at modulating gut microbiota, including dietary changes, probiotics and faecal microbiota transplantation. However, the role of gut microbiota in ADHD is still inadequately defined, necessitating larger studies to address the interaction in the GBA for a comprehensive understanding of molecular mechanisms. In addition, exploring this relationship holds promising implications for developing personalized therapeutic interventions and novel dietary strategies in order to mitigate ADHD symptoms.
